# Trehalose promotes *Rhodococcus* sp. strain YYL colonization in activated sludge under tetrahydrofuran (THF) stress

**DOI:** 10.3389/fmicb.2015.00438

**Published:** 2015-05-13

**Authors:** Zhixing He, Kai Zhang, Haixia Wang, Zhenmei Lv

**Affiliations:** College of Life Sciences, Zhejiang UniversityHangzhou, China

**Keywords:** trehalose, tetrahydrofuran, SDIMOs, CYP153, *Rhodococcus* sp. YYL

## Abstract

Few studies have focused on the role of compatible solutes in changing the microbial community structure in bioaugmentation systems. In this study, we investigated the influence of trehalose as a biostimulant on the microbial community in tetrahydrofuran (THF)-treated wastewater bioaugmentation systems with *Rhodococcus* sp. YYL. Functional gene profile changes were used to study the variation in the microbial community. Soluble di-iron monooxygenases (SDIMO), particularly group-5 SDIMOs (i.e., tetrahydrofuran and propane monooxygenases), play a significant role in the initiation of the ring cleavage of tetrahydrofuran. Group-5 SDIMOs genes are enriched upon trehalose addition, and exogenous tetrahydrofuran monooxygenase (*thmA*) genes can successfully colonize bioaugmentation systems. Cytochrome P450 monooxygenases (P450s) have a significant role in catalyzing the region- and stereospecific oxidation of non-activated hydrocarbons, and THF was reported to inhibit P450s in the environment. The CYP153 family was chosen as a representative P450 to study the inhibitory effects of THF. The results demonstrated that CYP153 family genes exhibited significant changes upon THF treatment and that trehalose helped maintain a rich diversity and high abundance of CYP153 family genes. Biostimulation with trehalose could alleviate the negative effects of THF stress on microbial diversity in bioaugmentation systems. Our results indicated that trehalose as a compatible solute plays a significant role for environmental strains under extreme conditions.

## Introduction

Bioaugmentation, which introduces certain functional microorganisms into indigenous bacterial populations, is an attractive environmental clean-up technique for reducing recalcitrant compounds in contaminated soil or water (Tyagi et al., 2011a). As a potentially powerful tool to direct community structure and metabolic capacities, the outcome of bioaugmentation is usually unpredictable (Falk et al., 2013) because maintaining persistent survival rates and activities of the introduced bacteria are the core and most difficult issues of bioaugmentation (El Fantroussi and Agathos, 2005). However, colonization is determined by many abiotic factors (e.g., temperature, pH-value, aeration, and nutrient content) and biotic factors (predation and competition) in remediation systems (Mrozik and Piotrowska-Seget, 2010). To help the introduced bacteria successfully colonize a new environment, biostimulation is always employed to enhance bioaugmentation efficiency (Arjoon et al., 2013; Mahanty et al., 2013; Fan et al., 2014). Biostimulation is the addition of nutrients to the system in order to improve the degradation of the microbial populations (Nikolopoulou and Kalogerakis, 2009). The biostimulants could be N and P (Delille et al., 2009; Singh et al., 2014), biosurfactants (Bordoloi and Konwar, 2009), or carbon sources (Sakultantimetha et al., 2011; Taccari et al., 2012).

Trehalose, as a compatible solute, could provide protection from dehydration, osmotic shock, extreme temperature, oxidative damage, and even radioactive damage (Crowe, 2007). Trehalose exerts its protective effects on living organisms by preventing protein denaturation, DNA damage and membrane fracture (Frederick et al., 2013). In one study, an engineered bacterium producing trehalose was more effective in reducing chromate than the wild-type strain (Frederick et al., 2013). Zhang and Van (2012) found that the trehalose concentration exhibited a significant correlation with desiccation-contributed die-off coefficients in soil *Escherichia coli* populations. In our previous study, the tetrahydrofuran (THF)-degrading bacterium *Rhodococcus* sp. strain YYL was found to accumulate trehalose during the THF degradation process (He et al., 2014). Despite these insights into cell mechanisms, trehalose has not been used as a biostimulant in bioremediation. Only Vyrides et al. (2010) has reported that another compatible solute, glycine betaine, enhanced saline synthetic sewage degradation and dissolved organic carbon (DOC) removal in a continuous submerged anaerobic membrane bioreactor (SAMBRs).

Bioaugmentation with the introduction of pollutant degrading-bacterial strains can improve the degradation of the pollutant but may also result in a change in the microbial community. For example, the inoculation of strain WBC-3 primarily affected the indigenous bacterial community structure during methyl parathion degradation (Wang et al., 2014). Diversity indices of a microbial community can be used to predict its function in natural and engineered environments (Smith et al., 2013; Seshan et al., 2014). Additionally, the metabolic potential of the other pollutants might be influenced by the pollutant in the bioremediation system.

Soluble di-iron monooxygenases (SDIMOs) are key multicomponent enzymes in the initial oxidation of hydrocarbons in phylogenetically and physiologically diverse bacteria, which could be divided into five groups according to component arrangement, substrate specificity, and sequence similarity (Coleman et al., 2006). The physiological roles of SDIMOs correspond to aromatic/alkene monooxygenases (group 1), phenol monooxygenases (group 2), soluble methane monooxygenases (group 3), alkene monooxygenases (group 4), and THF/ propane monooxygenases (group 5) (Leahy et al., 2003; Coleman et al., 2006). THF monooxygenase was responsible for converting THF into 2-hydroxytetrahydrofuran, which was the significant step in the THF degradation pathway (Masuda et al., 2012). Group-5 SDIMOs were correlated with 1, 4-dioxane in environmental samples (Li et al., 2013). THF and 1, 4-dioxane are both cyclic ethers; hence, it will be of interest in predicting THF degradation potential with the biomarker gene *SDIMO*.

THF is an inhibitor of cytochrome P450-dependent monooxygenases (P450s), which are versatile biocatalysts that catalyze the regio- and stereospecific oxidation of non-activated hydrocarbons under mild conditions (Moody, 1991; Urlacher and Girhard, 2012). The extensive P450 superfamily constitutes more than 1000 different enzymes, and the CYP153 family is related to alkane degradation in bacteria (Ji et al., 2013). Recent studies found that a wide range of bacteria contain *CYP153* genes, encoding cytochrome P450 alkane hydroxylase (Van Beilen et al., 2006). In this study, original activated sludge (OA) was obtained from the sewage treatment of a coking plant. CYP153 family genes were identified that were rich in OA. For the purpose of studying the inhibitory effects of THF on P450s, the *CYP153* gene was chosen as a representative P450 gene.

This study examines the effect of trehalose on the efficiency of bioaugmentation with the THF-degrading bacteria *Rhodococcus* sp. YYL and alleviating THF inhibitory effects on P450s in a batch reactor treating THF wastewater. The diversity, composition and abundance of the functional genes *SDIMO* and *CYP153* were synchronously investigated to study the effects of biostimulation on system functions.

## Materials and methods

### Reactor setup and operational conditions

To study the effects of trehalose on bioaugmentation systems with *Rhodococcus* sp. YYL, two identical lab-scale reactors with an effective volume of 2 L (height: inner diameter = 71.3 cm: 7.0 cm) were operated in a sequencing mode, treating synthetic THF wastewater containing 1.80 g NH_4_Cl, 0.81 g K_2_HPO_4_·3H_2_O, 0.40 g MgSO_4_·7H_2_O, 0.006 g ZnSO_4_·7H_2_O, and 0.024 g FeSO_4_·7H_2_O in 1 L of water adjusted to pH 8.20 by NaOH. THF was directly added into synthetic water with concentration of 20 mM, and the THF with 99% purity was obtained from China National Medicine Group (Shanghai, China). The two reactors were bioaugmented with strain YYL. For the experiments, 1% (v/v) bacteria (OD_600_ = 1.0) was inoculated into the reactor every 3 days; the third inoculation marked the end of the bioaugmentation phase. After bioaugmentation (BA), one reactor was operated as the control (non-trehalose BA reactor) and the other reactor was supplemented with 2 mM trehalose (trehalose BA reactor). The original activated sludge was derived from the sewage treatment system of a coke-over plant.

The reactors were operated at 25 ± 2°C, with 4–6 mg/L dissolved oxygen (DO). The duration of a complete cycle was 72 h and consisted of 4 phases: filling (15 min), operation (70 h), settling (1.5 h), and discharge (15 min). After settling, the supernatant in excess of 250 mL was discharged, and the reactors were then supplied with new synthetic wastewater. The reactors were studied for 96 days. The activated sludge was sampled at day 24, 48, 72, and 96. The samples were stored at −80°C for molecular analysis and 4°C for degradation experiments.

### Degradative activity of activated sludge in a shaking flask culture

Due to the high volatility of THF, its concentration at the end of a cycle in both reactors did not actually reflect the THF degradative ability of microorganisms in activated sludge. Hence, approximately 100 mL activated sludge in reactors at the sampling points were collected and cultured in a 500 mL shaking-flask with a rubber seal. Activated sludge was diluted to a 1.0 g/L MLSS concentration by synthetic wastewater and then supplemented with 20 mM THF. During cultivation, the agitation ratio was maintained at 140 rpm and the temperature was maintained at 25°C. The THF concentration was determined using gas chromatography after 72 h. Three replicates were conducted for every activated sludge sample.

### DNA extraction

Genomic DNA was extracted from sludge samples using a PowerSoil™ Kit (Bio 101, Carlsbad, CA, USA) according to the manufacturer's protocol. The DNA quality was examined by 1.0% (w/v) agarose gels and quantified with a NanoDrop ND-1000 spectrophotometer (NanoDrop Technologies, Wilmington, DE, USA).

### Denaturing gradient gel electrophoresis (DGGE) analysis

DGGE was used to assess the diversity of *SDIMO* and *CYP153* genes in activated sludge. For the *SDIMO* gene, a nested PCR strategy was used due to its low abundance. Two pairs of degenerate primers were designed according to the conservative sequences of the α-subunit of SDIMOs (Coleman et al., 2006). The expected length of the amplified fragment was 432 bp. One pair of primers was used to amplify 337 bp of the *CYP153* gene (Kim et al., 2007).

DGGE analysis was performed using a DCode™ Universal Mutation Detection System (Bio-Rad Laboratories, Hercules, CA, USA). The PCR products of *SDIMO* and *CYP153* genes were loaded onto 8% (w/v) polyacrylamide (37.5:1, acrylamide: bisacrylamide) gels in a 1 × TAE buffer (40 mM Tris, 20 mM acetate, 1 mM EDTA, pH 7.4) with a denaturing gradient of 30–60 and 30–70%, respectively. Electrophoresis was run at 60°C and 120 V for 12 h for *SDIMO*, and 10 h for *CYP153*. The gels were silver stained after electrophoresis following the protocol described by Bassam et al. (1991). Digital images of the gels were obtained by scanning, and the bands were excised and sequenced.

### Clone libraries and sequence analysis

The activated sludge at day 96 in the reactors was chosen to construct the *SDIMO* and *CYP153* gene clone libraries. PCR products were cloned using the pMD19-T vector (TaKaRa, Bio Inc., Shiga, Japan) according to the manufacturer's instructions. At least 20 positive clones for *SDIMO* genes and 60 positive clones for *CYP153* genes from each sample were selected for sequencing (Sangon Biotech, Shanghai, China). The sequences were edited using the DNAstar software package (DNAstar, USA). The sequences were confirmed online by NCBI BLAST and aligned using ClustalX (Version 1.81). The diversity was determined by rarefaction analysis using PHYLIP (Version 3.69). The sequences displaying more than 97% identity with each other were grouped into one operational taxonomic unit (OTU) by using the furthest neighbor algorithm in the DOTUR program (Schloss and Handelsman, 2005). The coverage of the clone libraries was calculated as previously described (Hill et al., 2003). All OTU representative sequences, their nearest neighbors and some reference sequences were analyzed by BLAST and imported in MEGA (Version 5) to construct unrooted phylogenetic trees using the neighbor-joining method. The relative confidence of the tree topologies was evaluated by performing 1000 bootstrap replicates.

### Real-time quantitative PCR (qPCR)

In this study, qPCR used to quantify the *SDIMO* and CYP153 genes and *thmA*. The gene *thmA* is a conserved region of the THF-degrading monooxygenase α-subunit gene of strain YYL, which catalyzed the significant step of THF degradation pathway from THF into 2-hydroxytetrahydrofuran. The special pair of primers was designed for specifically targeting the gene *thmA* in activated sludge, which was thmA-F:5′-GGTTGCCGTACTTATAGT-3′; thmA-R: 5′-GACACTTCTG TACCTCCT-3′.

qPCR was carried out using a Corbett Rotor-Gene 6000 (Qiagen, Hilden, Germany). The reaction was performed in 20 μL containing 10 μl SYBR® *Premix Ex Taq* (TaKaRa), 0.8 μL of each forward and reverse gene-specific primer (10 μM), 5 ng DNA sample and RNase and DNase free water to a final volume of 20 μL. The reactions for each sample were carried out in three replicates.

The one-point calibration method for absolute quantification was used to calculate gene abundance (Brankatschk et al., 2012). The PCR efficiency for each individual reaction was derived from the slope of the regression line fitted to a subset of baseline-corrected data points in the log-linear phase using the LinReg PCR program (v 11.4) (Ramakers et al., 2003). Then, the results were exported, and the mean PCR efficiency and C_t_ of each sample were calculated as the arithmetic mean of all replicates with the correlation factors (R^2^) never below 0.995.

## Results

### THF degradation activity of activated sludge from reactors

**Figure 1:**
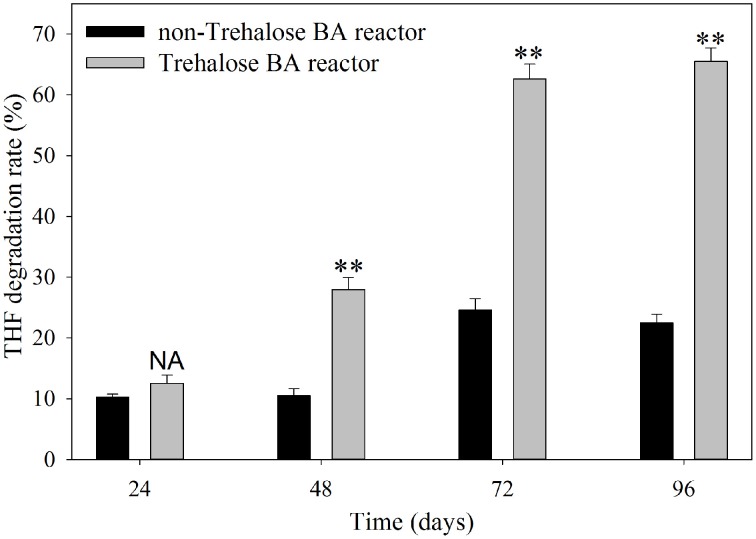
**THF degradation rate of activated sludge from a non-trehalose BA reactor and a trehalose BA reactor.** One-Way ANOVA analysis was used to analyze variances between the non-trehalose BA reactor and the trehalose BA reactor. NA, *P* > 0.05; ^**^P < 0.01.

The THF degradation rate in shaking-flask cultures of activated sludge are shown in Figure 1. The results indicated that the degradation rates of activated sludge from the non-trehalose BA reactor and the trehalose BA reactor increased from day 24 to 96, and the differences in THF degradation activity between the two reactors gradually increased over time. When the reactors were analyzed after 72 days, the THF degradation activities of activated sludge remained stable. This result suggested that successful colonization of strain YYL could be achieved in both reactors, but colonization efficiency was higher in the trehalose BA reactor than in the non-trehalose BA reactor. Hence, biostimulation with trehalose would be benifical for degradations strains colonizating in bioaugmentation systems.

### Changes in the diversity, composition, and abundance of the *SDIMO* gene

**Figure 2:**
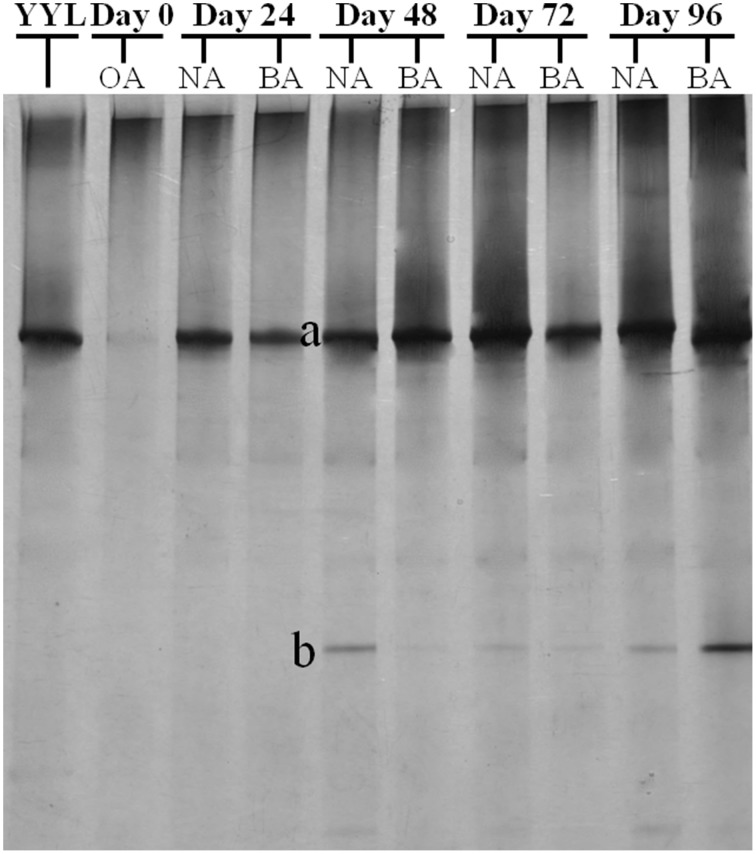
**DGGE fingerprinting profiles of the *SDIMO* gene from the activated sludge samples at different periods during reactor operation, indicating the change in microbial diversity and community composition.** YYL, represents the THF monooxygenases of *Rhodococcus* sp. YYL; OA, represents the original activated sludge; NA, represents the activated sludge from non-trehalose BA reactor; BA, represents the activated sludge from the trehalose BA reactor.

PCR-DGGE analysis was used to investigate the changes in the *SDIMO* gene diversity during the reactor operation period. The genotype richness, as indicated by two bands in the SDIMO-DGGE profile, was generally low in all activated sludge samples (Figure 2). A very weak band was detected in the original activated sludge (OA) due to low abundance of the *SDIMO* gene. Band a was the only band detected in strain YYL, suggesting that it should be representative of the THF monooxygenase gene (*thmA*). Sequence analysis further confirmed that band a belonged to a *Rhodococcus* sp. YYL THF monooxygenase (with a similarity of 99%) (Table 1). Band b was not detected in samples from the two reactors during the early operation period (day 24); it appeared after day 48 in activated sludge from both reactors treating THF wastewater. The sequence analysis of band b revealed that it was *Rhodococcus* sp. RR1 propane monooxygenase (with a similarity of 96%) (Table 1). The sequence similarity between band a and band b was 60.6%.

**Table 1:** **Sequence analysis of DGGE bands from Trehalose BA reactor and non-Trehalose BA reactor**.

**Band**	**Accession**	**Similarity (%)^a^**	**Closest organism in GenBank (Accession)**	**Phylogenesis^b^**
*CYP153*-a	KP306543	82	*Phenylobacterium zucineum* HLK1 (CP000747)	α-Proteobacteria
*CYP153*-b	KP306544	83	*Caulobacter crescentus* NA1000 (CP001340)	α-Proteobacteria
*CYP153*-c	KP306545	85	Uncultured bacterium cyp153 gene (HF561844)	Unclassified
*CYP153*-d	KP306546	80	Uncultured *Rhizobiales* bacterium (GU586463)	α-Proteobacteria
*CYP153*-e	KP306547	88	*Rhodopseudomonas palustris* (CP000301)	α-Proteobacteria
*CYP153*-f	KP306548	86	*Parvibaculum lavamentivorans* (CP000774)	α-Proteobacteria
*CYP153*-g	KP306549	81	Uncultured bacterium (HF561665)	Unclassified
*CYP153*-h	KP306550	91	Uncultured bacterium clone C7-48 (KF548263)	Unclassified
*CYP153*-i	KP306551	83	*Parvibaculum* sp. S13-5 (FJ218204)	α-Proteobacteria
*CYP153*-j	KP306552	85	*Phenylobacterium zucineum* HLK1 (CP000747)	α-Proteobacteria
*CYP153*-k	KP306553	80	*Parvibaculum* sp. S13-5 (FJ218204)	α-Proteobacteria
*CYP153*-m	KP306554	88	*Parvibaculum hydrocarboniclasticum* (GU904008)	α-Proteobacteria
*CYP153*-n	KP306555	82	*Bradyrhizobium japonicum* (BA000040)	α-Proteobacteria
*CYP153*-o	KP306556	81	*Parvibaculum lavamentivorans* DS-1 (CP000774)	α-Proteobacteria
*CYP153*-p	KP306557	82	Uncultured bacterium P450 (AB206804)	Unclassified
*CYP153*-q	KP306558	85	*Parvibaculum lavamentivorans* DS-1 (CP000774)	α-Proteobacteria
*CYP153*-r	KP306559	88	*Parvibaculum* sp. S18-4 (FJ218239)	α-Proteobacteria
*CYP153*-s	KP306560	80	*Rhodococcus* sp. DEE5301 (DQ847174)	Actinobacteridae
*CYP153*-t	KP306561	84	Uncultured bacterium cyp153 gene (HF561756)	Unclassified
*CYP153*-u	KP306562	80	Uncultured bacterium P450 (AB206804)	Unclassified
*CYP153*-v	KP306563	88	*Parvibaculum hydrocarboniclasticum* (GU904008)	α-Proteobacteria
*CYP153*-w	KP306564	80	Uncultured *Rhizobiales* bacterium (GU586482)	α-Proteobacteria
*CYP153*-x	KP306565	91	Uncultured bacterium clone C7-48 (KF548263)	Unclassified
*SDIMOs*-a	KP306541	99	*Rhodococcus* sp. THF monooxygenases. YYL (EU732588)	Actinobacteridae
*SDIMOs*-b	KP306542	97	*Rhodococcus* sp. RR1 propane monooxygenase hydroxylase (HM209445)	Actinobacteridae

a Similarity: The similarity between the sequences from DGGE and their closest relatives in GenBank.

b Phylogenesis: Phylogenetic groups of the DGGE bands matcher in GenBank.

**Figure 3:**
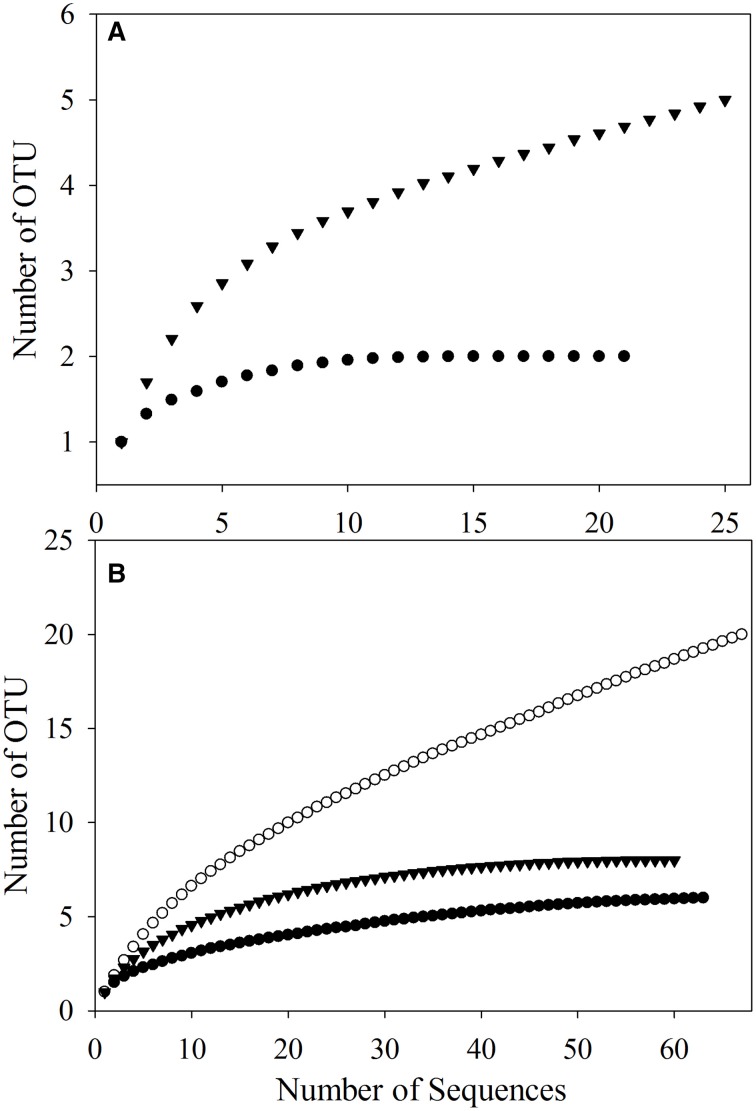
**Rarefaction analysis of *SDIMO* gene sequences (A) and *CYP153* gene sequences (B) obtained from activated sludge samples at day 96.** Both genes were defined at 97% sequence similarity. The white dot represents the original activated sludge; the black dot represents the activated sludge from the non-trehalose BA reactor; the black triangle represents the activated sludge from the trehalose BA reactor.

To better study the *SDIMO* gene diversity difference between non-trehalose BA reactor activated sludge (NA) and trehalose BA reactor activated sludge (BA), two clone libraries (activated sludge of both reactors at day 96) were constructed using the PCR products of the *SDIMO* gene. The rarefaction curve of the BA clone library was always above that of the NA clone library, indicating that the *SDIMO* gene richness of BA was higher than that of NA (Figure 3A). DOTUR analyses of SDIMO gene clone libraries allowed the definition of 6 OTUs from 46 quality sequences in activated sludge samples using a 97% sequence similarity cut-off, and the clones were grouped into 2 and 5 OTUs at NA and BA, respectively. Diversity indices (abundance-based coverage estimator [ACE], Chao1 and Shannon) and the coverage for individual libraries were estimated (Table 2). The coverage of the clone libraries was above 90%. BA exhibited a higher diversity (Shannon = 1.28, Chao1 = 7 and ACE = 7.40) than NA (Shannon = 0.49, Chao1 = 2, and ACE = 2). These results were consistent with the PCR-DGGE and rarefaction curve analyses and suggested that trehalose was conducive to *SDIMO* gene diversity in THF-treated wastewater systems.

**Table 2:** **OTU numbers and diversity indexes of *SDIMO* and *CYP153* genes in the clone libraries of activated sludge samples**.

**Genes**	**Samples**	**No. of sequences**	**No. of OTUs**	**Coverage**	**Shannon**	**Chao1**	**ACE**
*SDIMO*	NA	21	2	1.00	0.49	2	2
	BA	25	5	0.92	1.28	7	7.34
*CYP153*	OA	67	20	0.85	2.43	42	47.58
	NA	63	6	0.99	1.03	6	6.57
	BA	60	8	1.00	1.61	8	8

**Figure 4:**
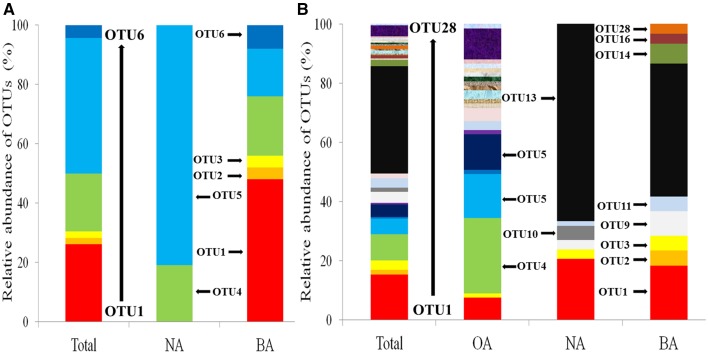
**Relative abundances of OTUs of *SDIMO* gene (A) and *CYP153* gene (B) in all clone libraries combined (Total) and in the individual clone library of activated sludge samples.** OA, represents the original activated sludge; NA, represents the activated sludge from the non-trehalose BA reactor; BA, represents the activated sludge from the trehalose BA reactor.

Phylogenetic analysis allowed the identification of the *SDIMO* gene group (Figure S1). OTU1 and OTU2 were clustered with *prmA*, encoding propane monooxygenase, from *Pseudonocardia dioxanivorans* CB1190 or *Pseudonocardia* sp. TY-7. OTU3 shared 98% similarity with propane monooxygenase of *Rhodococcus* sp. RR1. All three OTUs were unique in BA, which represented 52% of all the *SDIMO* genes in BA (Figure 4A). The other OTUs (OTU4, OTU5, and OTU6) were clustered into one group, identified as THF monooxygenase of *Rhodococcus* sp. YYL, which contributed 100% of the *SDIMO* genes in NA and 48% in BA (Figure 4A).

**Figure 5:**
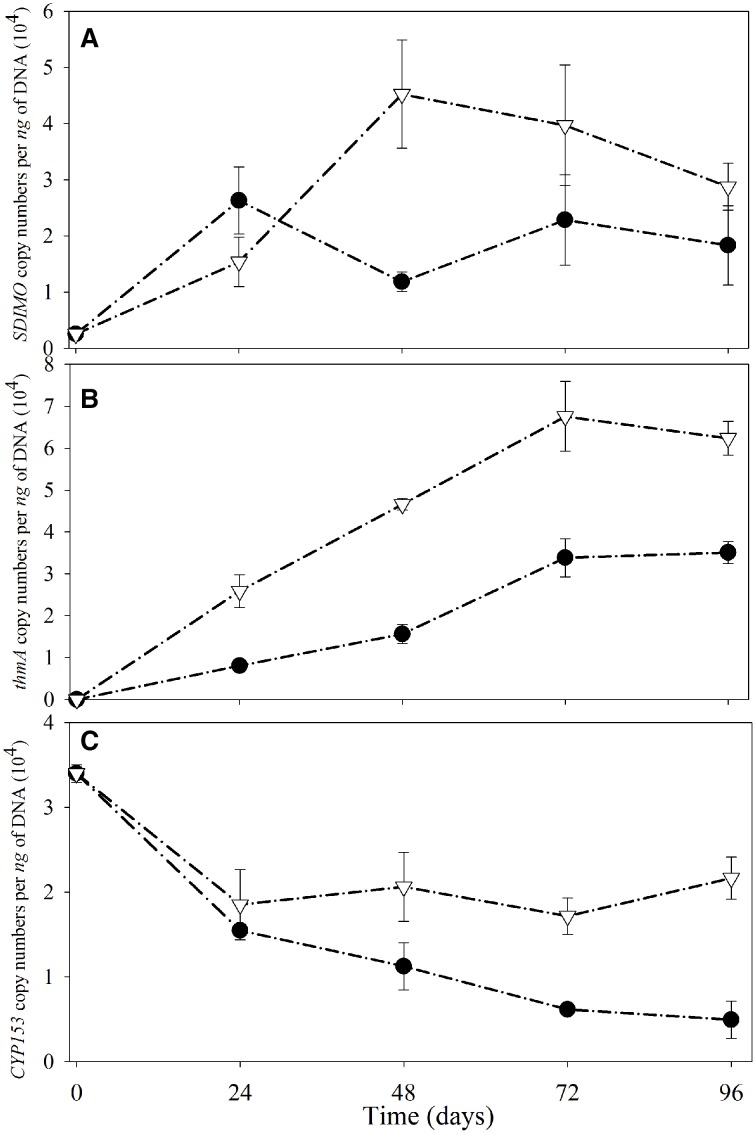
**Quantitative analysis of *SDIMO* (A), *thmA* (B), and *CYP153* genes (C) from activated sludge samples at different periods during reactor operation.** The black dot represents the non-trehalose BA reactors; the white triangle represents the trehalose BA reactors.

The *SDIMO* gene copy numbers per ng of DNA are shown in Figure 5A. The abundance of *SDIMO* genes ranged from 1.18 × 10^4^ to 2.63 × 10^4^ copies per ng of DNA, and the highest was observed at day 24 in the NA reactor. However, the highest abundance of *SDIMO* genes in the trehalose BA reactor was detected with 4.52 × 10^4^ copies per ng of DNA at day 48, and the lowest was 1.54 × 10^4^ copies per ng of DNA at day 24. *SDIMO* gene expression increased due to trehalose biostimulation. The abundance of the *thmA* gene kept increasing in the first 72 day in both reactors, and the rate was always higher in the trehalose BA reactor (from 0 to 6.76 ×10^4^ copies per ng of DNA) than in the non-trehalose BA reactor (from 0 to 3.39 × 10^4^ copies per ng of DNA) (Figure 5B). In conclusion, the reactors being biostimulated with trehalose possessed of higher diversity and abundance of *SDIMO* gene. Besides, trehalose not only be benifical for THF monooxygenase gene but also for propane monooxygenase gene in the reactor.

### Changes in the diversity, composition, and abundance of the *CYP153* gene

**Figure 6:**
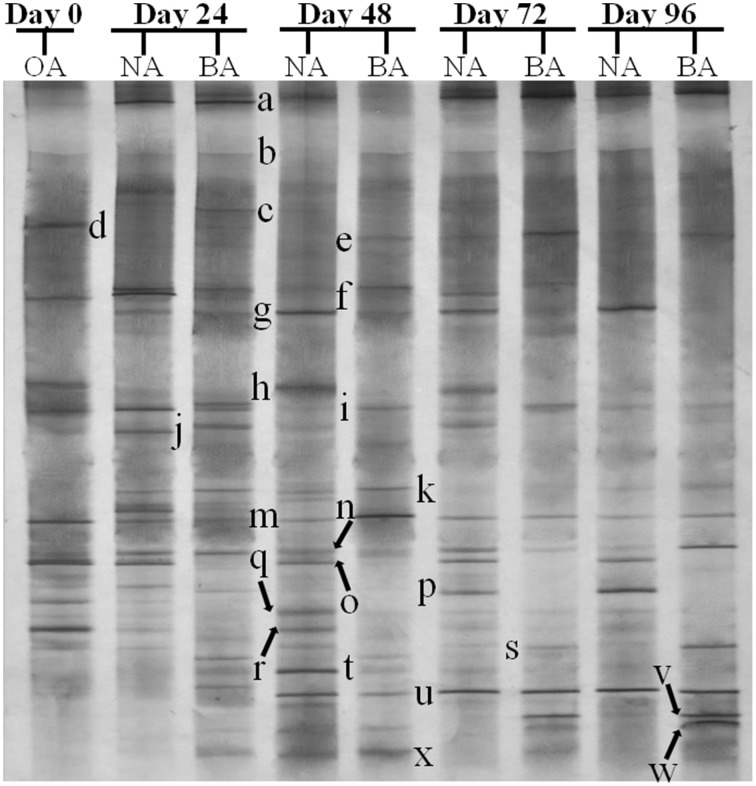
**DGGE fingerprinting profiles of the *CYP153* gene from the activated sludge samples at different periods during reactor operation, indicating the change in microbial diversity and community composition.** OA, represents the original activated sludge; NA, represents the activated sludge from non-trehalose BA reactor; BA, represents the activated sludge from the trehalose BA reactor.

**Figure 7:**
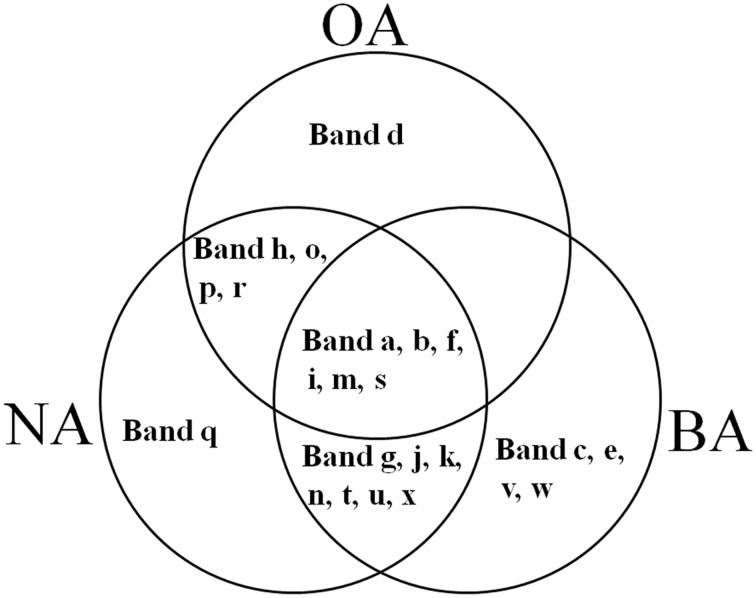
**Venn diagram showing the shared and unique bands detected in the *CYP153* gene DGGE profile of activated sludge.** OA, represents the original activated sludge; NA, represents the activated sludge from the non-trehalose BA reactor; BA, represents the activated sludge from the trehalose BA reactor.

CYP153, a family of cytochrome P450 monooxygenases, was recently discovered in alkane-degrading bacteria (Van Beilen et al., 2006). Dynamic changes of *CYP153* gene diversity should be impacted by THF in THF-treated wastewater systems. The DGGE profile indicated that the *CYP153* gene was abundant in activated sludge and significant changes occurred during the reactor operation period (Figure 6). As shown in Figure 6, six bands (a, b, f, i, m, and s) appeared in OA, NA and BA, and bands a, b, i, and m appeared in all activated sludge samples. However, band f was not observed in NA and BA at day 96 and band s was only detected in BA at day 96. Bands g, j, k, n, t, u, and x were only detected in NA and BA and not in OA, indicating that the abundance of these bands was increased in activated sludge as the operation process increased. Bands k, n, and u were detected in both NA and BA, while bands j and t were lost in both NA and BA, and bands g and k were only detected in NA and BA at day 96 (Figures 6, 7). In addition, bands h, o, p, and r were visualized in OA and NA but not in BA, and bands o and p were detected in NA at day 96 (Figures 6, 7). However, no bands were found that existed in OA and BA but not in NA (Figures 6, 7). Only one band was unique to OA (band d) or NA (band q), but four bands (c, e, v, and w) were unique to BA (Figures 6, 7). These bands were all sequenced, and the alignment results showed that these sequences shared moderate identities (80–91%) with the *CYP153* gene in GenBank (Table 1).

A total of 190 clones were obtained from the combined *CYP153* gene clone libraries (67 clones in OA, 63 clones in NA and 60 clones in BA at day 96). Rarefaction analysis yielded asymptotic curves in NA and BA at the distance level of 0.03, but the rarefaction curve of OA tended to be linear (Figure 3B). The coverage index of clone libraries was 0.85, 0.99, and 1.00 in OA, NA and BA, respectively (Table 2). The sequences were grouped into 28 OTUs (3% cutoff) by DOTUR, and only 3 OTUs were shared in the three clone libraries. For each clone library, 20 OTUs, 6 OTUs, and 9 OTUs were identified among 67 sequences in OA, 63 sequences in NA and 60 sequences in BA, respectively (Table 2). In addition, the order of diversity indices (ACE, Chao 1 and Shannon) was as follows: OA>BA>NA (Table 2).

The relative abundance of OTUs in each clone library is shown in Figure 4B. The total percentage of the three shared OTUs (OTU1, OTU3, and OTU11) contributed 11.9, 25.4, and 28.3% in OA, NA and BA, respectively. OTU4 was the most abundant in OA, which represented 25.4% of *CYP153* genes (Figure 4B). Phylogenetic analysis showed that OTU4 shared 90% similarity with *Parvibaculum* sp. S18-4 (FJ218239) (Figure S2). OTU13 was the major percentage of *CYP153* genes in both NA and BA (Figure 4B), which shared a low similarity (<80%) with *CYP153* genes in GenBank. It was not detected in the OA clone library. The cluster results also indicated that the CYP153 family was not conserved and has a high diversity (Figure S2).

Quantification of the *CYP153* gene illustrated that THF has a negative effect on *CYP153* gene abundance (Figure 5C). The highest abundance of the *CYP153* gene was observed at day 0 in both reactors. A decreasing trend was found from day 0 to day 96 in the non-trehalose BA reactor; however, the abundance only decreased at 24 d and small fluctuations continued after 24 day in the trehalose BA reactor. Trehalose could be beneficial to maintain *CYP153* gene abundance in THF-treated wastewater systems. In conclusion, trehalose alletivated THF stress on microbial community owning CYP153 gene, resulting in higher gene diversity and abundance.

## Discussion

Bioaugmentation has been recognized since the 1970s as a straightforward technology to remove recalcitrant pollutants from the environment in cases where specific degrading organisms are lacking (Gertler et al., 2009; Bai et al., 2011). However, bioaugmentation is not always efficient due to low survival rates and the degradation efficiency of the introduced inocula (Tyagi et al., 2011b). Biostimulation is often additionally used as a strategy to modify the environment for the successful colonization of introduced inocula (Nikolopoulou and Kalogerakis, 2009).

In this study, trehalose was adopted as a biostimulant and was found to benefit strain YYL colonization in THF-treated wastewater systems depending on the results of the THF degrading activity of the activated sludge and *thmA* abundance. Trehalose accumulation in strain YYL plays a significant role in the process of THF degradation (He et al., 2014). Thus, we propose that exogenous trehalose might help strain YYL adapt to the new environment under THF stress. This is the first report of trehalose as a biostimulant to help bioaugmentation. Some previous studies have reported that trehalose acts as a compatible solute in environmental bacterial strains (Pade et al., 2012; Reina-Bueno et al., 2012). In addition, trehalose concentration has a strong correlation with environmental stresses (Zhang and Van, 2012). Another type of compatible solute, glycine betaine, could enhance saline synthetic sewage degradation and DOC removal in continuous SAMBRs (Vyrides et al., 2010). Our study further indicated that some compatible solutes have application potential in bioremediation.

Successful colonization of introduced inocula could pose alterations in microbial communities in bioaugmentation systems (Wang et al., 2014). Most studies evaluated the whole community variation according to the bacterial 16S rRNA genes (Yao et al., 2012; Wang et al., 2014). For some specific pollutants, functional genes were of significant interest as biomarkers for studying potential degrading-bacterial communities (Acosta-Gonzalez et al., 2013; Penton et al., 2013). There was no biomarker gene for predicting THF degradation in environmental samples. The reason may be that only three genera of bacteria (*Rhodococcus* sp., *Pseudonocardia* sp., and *Pseudomonas* sp.) are known to degrade THF. However, 1, 4-dioxane, with a closely similar structure to THF, was correlated with *SDIMO* in other studies (Li et al., 2013; Gedalanga et al., 2014). In this study, bioaugmentation of strain YYL resulted in *SDIMO* being detected in BA and NA. Meanwhile, the abundance and diversity richness of *SDIMO* increased in both bioaugmentation systems treating THF wastewater. However, biostimulation with trehalose helped BA not only owing to higher *SDIMO* abundance (Figure 3A) but also by increasing *SDIMO* diversity (Figure 4A) compared to NA. Trehalose might help microorganisms to adapt to THF stress. In addition, the abundance of a propane monooxygenase gene increased with the increasing operational processes under THF stress in both NA and BA. Expression of the propane monooxygenase gene was time dependent with 1, 4-dioxane biodegradation (Gedalanga et al., 2014). Thus, biodegradation of THF might be correlated with the propane monooxygenase gene in activated sludge. This might explain why THF and propane monooxygenase genes are always found in one bacterial strain simultaneously (Sales et al., 2013).

THF are inhibitors of cytochrome P450s in microorganisms and affect the microbial community of activated sludge (Urlacher and Girhard, 2012; Yao et al., 2012). However, P450s make up one of the largest superfamilies of heme-containing enzymes (Kubota et al., 2005). It was difficult to evaluate THF-inhibiting effects on the entire P450 family. In this study, the original activated sludge (OA) was obtained from the treated sewage of a coking plant and was rich with alkane-degrading bacteria. CYP153, one family of cytochrome P450, was recently discovered in alkane-degrading bacteria (Van Beilen et al., 2006). Thus, *CYP153*, as a soluble alkane-degrading gene in bacterium, was chosen as the representative family for P450 to evaluate THF inhibitory effects on P450s. Depending on the DGGE profile, there was a high diversity of the *CYP153* gene in activated sludge. Richness in diversity may be attributed to a universal high horizontal gene transfer potential of *CYP153* genes in Gram-positive alkane-degrading actinomycetes (Nie et al., 2014). In addition, DGGE profiles and Venn diagrams (Figures 6, 7) revealed large changes in *CYP153* gene structure in activated sludge during the operational period, meaning that THF-treated bioaugmentation systems affected *CYP153* genes. This is the first report evaluating THF inhibition effects on P450s from the perspective of ecology.

Trehalose biostimulation could affect *CYP153* gene structure. In addition, diversity indices and abundances of *CYP153* were much higher in trehalose biostimulation systems than that in non-trehalose biostimulation systems at day 96. This diversity can greatly affect major ecosystems services such as pollutant removal (Hernandez-Raquet et al., 2013). Generally, the more richness in diversity and abundance of genes under stress, the less negative the effect of the stress (Kelly et al., 2013). These results demonstrated that exogenous trehalose biostimulation could help the bacterial strains possessing *CYP153* genes growing under THF stress. The reason for trehalose alleviating effects on *CYP153* genes may be: (1) trehalose entered into the bacterial strains possessing *CYP153* genes to resist THF stress; or (2) trehalose helped strain YYL to colonize in activated sludge, which resulted in decreasing THF, and a low concentration of THF posed less negative effects on the bacterial strains in activated sludge.

## Conclusion

In summary, bioaugmentation of strain YYL was successful in activated sludge under THF treatment, resulting in *SDIMO* genes being detected in activated sludge samples from two reactors and large changes in *CYP153* structures at different periods during reactor operation. However, bioaugmentation with trehalose as a biostimulant could lead to a much higher diversity and abundance of other degradative genes and help strain YYL, owing to higher quantities and THF degradation activity in activated sludge.

### Conflict of interest statement

The authors declare that the research was conducted in the absence of any commercial or financial relationships that could be construed as a potential conflict of interest.
